# Polymer waste and pollution in oral healthcare clinics: a systematic review

**DOI:** 10.1038/s41405-025-00342-8

**Published:** 2025-05-25

**Authors:** Anne Margrete Gussgard, Asbjørn Jokstad

**Affiliations:** Department of Clinical Dentistry, Faculty of Health Sciences, UiT The Arctic University of Norway, Tromsø, Norway

**Keywords:** Continuing professional development in dentistry, Dental materials, Occupational health, Infection control in dentistry

## Abstract

**Background:**

Modern oral healthcare extensively uses polymer items and devices derived from various monomeric compounds. These materials are essential for personal protective equipment, infection barriers, packaging, and intraoral devices. The COVID-19 pandemic has led to an increased reliance on single-use polymer items, causing supply chain disruptions and higher costs. This systematic review explores the extent of polymer waste and pollution generated in oral healthcare clinics.

**Materials and methods:**

A systematic review protocol was registered with PROSPERO and was formatted according to PRISMA guidelines and SWiM recommendations. Eligibility criteria included studies that provided quantified estimates of polymer waste or pollution in air or wastewater from oral healthcare clinics. Comprehensive electronic searches were conducted across several bibliometric databases, followed by data extraction and risk of bias assessments performed by two independent reviewers.

**Results:**

A total of thirty studies were included in the review. Sixteen papers reported on waste audits that detailed polymer waste data, while eight studies focused on pollution caused by polymer nano- and microparticles in clinical settings. Additionally, six experimental studies investigated potential leakage of monomeric eluates or polymer particles from landfill waste. There was significant variation in the amount of polymer waste generated per patient, ranging from 81 to 384 g per operatory room per day. On-site sampling revealed the presence of polymer nano- and microparticles in the clinic air, which was influenced by dental procedures and the equipment used.

**Conclusions:**

This review highlights critical knowledge gaps about polymer waste and pollution in oral healthcare clinics. The variability of study designs limited the feasibility of meta-analysis. Current evidence indicates substantial polymer waste generation, particularly from single-use items, as well as potential environmental impacts from monomeric eluates and polymer microparticles. Future research should focus on sustainable polymer waste management solutions to reduce environmental pollution in oral healthcare settings.

## Introduction

Modern oral healthcare relies on a wide range of polymer items and devices (PIDs) composed of monomeric compounds. Some compounds are used in personal protective equipment (PPE), as barriers to prevent cross-infection, and for packaging and wrapping. Other compounds have been explicitly designed to resist degradation in the patient’s mouth. A third category of polymer compounds is elastomers, which are employed as impression materials for creating casts (Supplementary Table 1).

Many devices for intraoral use are customised from monomeric materials in the clinic and polymerise chemically after mixing or following exposure to heat or light. The extent of remaining unreacted monomeric compounds varies depending on the degree of polymerisation. The properties of polymers, which can range from rigid to flexible and durable to fragile, are influenced by the presence of intended or unintended monomeric compounds and other copolymers. In this paper, ‘polymer’ comprises plastic and rubber-like materials, regardless of their content of unreacted monomeric compounds.

During the COVID-19 pandemic, the use of single-use polymer items increased significantly, leading to disrupted supply chains and rising costs [1]. The situation stimulated initiatives to improve the conservation of devices and instruments [2]. There is also a recognition of the importance of managing biomedical waste effectively, particularly hazardous waste containing infectious and potentially infectious materials [3]. Developing innovative solutions is crucial for facilitating the reprocessing and recycling of PIDs and other polymer products for circular use, as well as enhancing the current management of polymer waste from oral healthcare clinics [4].

There is growing recognition in healthcare clinics of the importance of reducing, reusing, and recycling monomeric materials and PIDs [5]. However, identifying the most effective and safe solutions remains challenging due to significant knowledge gaps. These include limited understanding of the potential hazards of nano- and microparticles (NMPs) and chemical additives leaching from medical plastics [6]. There is also insufficient evidence on best practices for segregating biomedical and domestic waste in hospitals and how to manage waste effectively to prevent NMP pollution in air and wastewater systems [7]. On a broader scale, the environmental impacts of monomeric eluates and polymer-derived NMPs in marine ecosystems are not fully understood [8–10]. Furthermore, the relative viability and long-term effectiveness of local versus centralised bioremediation strategies for polymer waste remain uncertain [11]. Finally, emerging research suggests potential risks to human health and the environment from persistent organic pollutants released by monomeric compounds [12, 13], including possible epigenomic effects—yet much remains to be explored.

The extent of polymer waste and pollution in oral healthcare clinics has not been systematically reviewed. This systematic review aims to address this knowledge gap by evaluating the existing scientific evidence to answer the research question: ‘How much polymer waste and pollution is generated in oral healthcare clinics?’

## Methods

Before beginning the work, we registered a protocol with the International Prospective Register of Systematic Reviews (PROSPERO CRD42023472616). The protocol serves as the foundation for this SR, formatted according to PRISMA (Preferred Reporting Items for Systematic Reviews and Meta-Analyses) [14], complemented by the Synthesis Without Meta-analysis (SWiM) recommendations [15].

### Eligibility criteria

We searched for studies that reported quantified estimates of polymer waste or pollution in the air or wastewater within oral healthcare clinics. A broad search was adopted since the topic is under-researched, with no limitations regarding variables such as the waste collection year, the timeframe for collecting waste, or the clinic setting, i.e., private or public single or group practices, hospitals, or faculty hospitals. The same applied to studies detailing the levels of monomeric eluate, monomeric degradation compounds, or NMPs associated with the collection, management, and disposal of polymer waste. Eligible studies included waste audits and field-based research reporting polymer pollution in oral healthcare clinics. Additionally, we included laboratory studies experimentally designed to estimate polymer pollution from waste collection, management, and disposal in oral healthcare clinics. Our only exclusion criterion was studies that did not report quantified estimates, regardless of study design, although the reference lists were routinely scrutinised to identify possible eligible studies.

### Information sources and search strategy

Two reviewers independently conducted systematic and comprehensive electronic searches to identify potentially eligible records. A Boolean search strategy was developed for searches in Pubmed: (Dentist OR dental health services[mesh] OR Dentistry[mesh]) AND (Polymers[mesh] OR Organic Chemicals[mesh] OR plastic*[tw] OR polymer*[tw] OR Resin*[tw] OR acryl*[tw]) AND ((Medical Waste[MeSH] OR Medical Waste Disposal[MeSH] OR Waste Management[MeSH] OR Waste Disposal Facilities[MeSH] OR Hazardous Waste[MeSH] OR Environmental Pollution[MESH] OR Dental Waste[MESH])). The SRA Polyglot Search Translator was adopted to modify the search strategy to various bibliometric databases and grey literature. Scientific literature was searched on the Cochrane Library, Embase via Ovid, EBSCOhost (restricted to CINAHL, Risk Management Reference Center, GreenFILE, MEDLINE, the eBook Open Access Collection, and AMED [The Allied and Complementary Medicine Database]), the National Library of Medicine (MEDLINE via PubMed), ScienceDirect, and the Web of Science (Supplementary Table 2).

Grey literature was explored through Google Scholar and the Abstracts Database of the International Association for Dental Research (IADR). The ProQuest Dissertations & Theses Global database was searched for master’s and doctoral theses. The language was limited to English. The reference lists of the included studies were further screened to identify additional relevant studies. The final search in the bibliometric databases was conducted in March 2025. Records from the literature searches were exported into EndNote, where duplicates were removed before being transferred to a relational database (Microsoft Access) to create a digital entry form for further data extraction from the primary studies.

### Selection and data collection process

One investigator (AJ) scrutinised the titles and abstracts of the identified records to ascertain whether the publication contained pollution estimates or polymer waste. Conversely, the second investigator (AMG) ensured that the excluded titles did not meet this criterion. In cases of disagreement, a consensus was reached on which articles to review in full text. The two investigators independently extracted data from eligible studies into an online Microsoft Access database form. The extracted data were compared, and any discrepancies were resolved through discussion. After confirming its accuracy, one investigator (AJ) entered the agreed-upon data into Access. The authors of the included studies were not contacted to verify missing data.

### Data items

Data were collected regarding the study objectives and methodology, as well as the relevant characteristics of the objects or study participants, interventions, sampling and measurement methods, and the duration of the study. The eligible outcomes and measures were as follows:

1. Polymer waste qualities or quantities: monomeric eluate, NMP count or mass per intervention, patient, or clinic measured daily, weekly, monthly, or according to any other time frame.
2. Polymer pollution qualities or quantities: monomeric eluate, NMP count or mass in the clinic’s ambient air or wastewater.
3. Monomeric eluate or NMP count in waste landfills associated with PIDs from oral healthcare clinics.

### Study risk of bias assessment

Exhaustive literature searches did not identify validated tools for addressing the risk of bias in waste audits. Consequently, we developed a checklist that included recommended processes mandated by the Environment Agency in the United Kingdom for the treatment and transfer of healthcare waste [16]. (Supplementary Table 3). Discrepancies between the two investigators’ assessments of bias risk were to be resolved by consensus, involving an evaluation of the risk of bias researcher if necessary.

### Effect measures

We planned to analyse the effect size of reported comparative approaches for reducing polymer waste, monomeric eluate, and polymer NMP content in air or wastewater. We intended to compare mean differences alongside estimated confidence intervals for aggregated continuous data. The waste audit data were to be converted into polymer waste mass per patient (g), operatory per day (kg) and operatory per year (kg). The heterogeneity of the study sample was assessed using I² statistics to determine the suitability of conducting a meta-analysis.

### Synthesis of outcome results

We aimed to synthesise effect estimates to obtain summary estimates using RevMan v.5.4, if comparable data could be identified in at least five studies with low clinical heterogeneity and statistical heterogeneity, as assessed by I² statistics. Data reported in measurement units that differed from other studies were transformed to facilitate comparisons within each synthesis. If meta-analyses were not feasible, we planned to follow the SWiM guidelines [17, 18]. We did not intend to conduct any analyses to investigate heterogeneity or to perform sensitivity analyses to evaluate the robustness of the synthesised results. Nor did we plan to assess the risk of bias resulting from missing results in a synthesis due to potential reporting biases, or to evaluate the certainty or confidence in the body of evidence for any outcomes.

### Certainty assessment

The GRADE (Grading of Recommendations, Assessment, Development, and Evaluations) framework was to be considered, provided sufficient outcome data could be extracted from the primary studies to undertake meta-analyses.

## Results

### Study selection

**Fig. 1:**
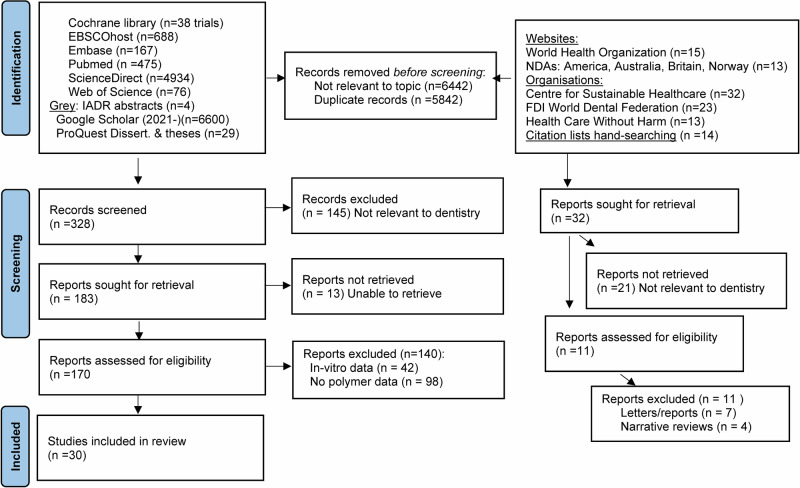
PRISMA flowchart detailing the number of records, full-text articles, and studies included at each stage of the systematic review process.

The bibliometric searches yielded numerous duplicates and records unrelated to the topic. After screening and thoroughly reading the full texts to assess eligibility, we identified 30 references for data extraction (Fig. 1).

Sixteen papers described waste audits that included data on polymer waste [5, 19–33]. Eight studies described polymer NMP pollution in the clinic work area [34–41]. Six experimental laboratory studies reported the potential leakage of monomeric eluates or polymer NMPs from landfill waste [42–47].

### Polymer waste

Waste generation and management in oral healthcare clinics is a recurring topic in national and international dental and medical journals. However, the number of research papers detailing the qualities and quantities of polymer waste and other waste remains limited. Few waste audits have documented specific amounts and these originate from Iran (*n* = 6) [21–23, 25, 27, 29], Greece (*n* = 3) [20, 26, 28], United Kingdom (*n* = 2) [5, 24], the USA (*n* = 2) [31, 32], and single studies from Australia [33], and Turkey [19]. Relevant supplementary data is a waste audit conducted in a hospital in Australia that identified the most common wasted polymers in surgical operating theatres [30] (Table 1).

**Table 1:** Waste audits that include polymer waste data.

Study	Country	Objectives	Methodology
Yeoh et al. [33]	Australia, Cairns	To analyse dental clinical waste in a university clinic setting to explore opportunities for sustainable practices.	Waste audit in clinic
Oxborrow et al. [32]	USA, Washington	To conduct a waste audit to identify areas of potential waste reduction and recommend interventions to reduce waste.	Waste audit in preclinic
Martin et al. [5]	U.K., Sheffield	To quantify (by number and mass) single-use items waste generated from oral healthcare in primary and secondary care clinical dental settings in the U.K.	Waste audit in clinic
Alstrom [31]	USA, Nebraska	To acquire data regarding waste management for a practice and plan for future improvement of waste management practice	Waste audit in clinic
Wyssusek et al. [30]	Australia, Brisbane	To measure the volume of polyethylene terephthalate and other polymers that are accumulated across operating rooms at a hospital in approximately one month	Waste audit in hospital
Aghalari et al. [29]	Iran, Babol	To investigate the quantity, quality and management of wastes in general and specialised dental offices in Babol, Mazandaran Province	Waste audit in clinic
Voudrias et al. [28]	Greece, Xanthi	To compare the composition and production rate of Greek dental solid waste produced by three dentist groups of Xanthi, Greece	Waste audit in clinic
Momeni et al. [27]	Iran, Birjand	To assess dental waste production rate and composition and approaches used to manage these waste products in 2017 in Birjand, Iran	Waste audit in clinic
Mandalidis et al. [26]	Greece, Xanthi	To determine the composition, characterisation and production rate of Greek dental solid waste	Waste audit in clinic
Majlesi et al. [25]	Iran, Qaem Shahr	To analyse the production of waste in dental offices of Qaem Shahr city.	Waste audit in clinic
Richardson et al. [24]	U.K., Plymouth	To use an audit approach to measuring the nature and quantity of clinical waste and assessing the feasibility of measuring the financial costs and potential carbon savings in the management of clinical waste	Waste audit in clinic
Amouei et al. [23]	Iran, Babol	to evaluate the quantity and composition of dental waste produced by general and specialised dental offices in Babol City.	Waste audit in clinic
Nabizadeh et al. [22]	Iran, Gorgan	To investigate solid waste production and its management in dental clinics in Gorgan, northern Iran.	Waste audit in clinic
Nabizadeh et al. [21]	Iran, Hamadan	To identify the components, composition and production rate of dental solid waste and associated management practices in dental offices in Hamadan, Iran	Waste audit in clinic
Kizlary et al. [20]	Greece, Xanthi	To determine the composition and production rate of dental solid waste produced by dental practices in the Prefecture of Xanthi, Greece	Waste audit in clinic
Ozbek et al. [19]	Turkey, Ankara	To identify the components of typical dental solid waste, variations in the composition between different clinics and management practices in the School of Dentistry at Hacettepe University in Turkey.	Waste audit in clinic

#### Study characteristics

Waste audits conducted between 2002 and 2023 demonstrate various approaches and contexts in which polymer waste is evaluated. Clinical settings include dental faculty clinics (*n* = 2) [19, 33], dental faculty undergraduate preclinic (*n* = 1) [32], private clinics (*n* = 5) [20, 22, 26, 28, 31], public clinics (*n* = 2) [25, 27], mixed clinics (*n* = 2) [5, 24], speciality clinics (*n* = 2) [21, 23, 29], and one surgical operating theatre [30]. Sample sizes differ markedly, with the most significant number of treated patients totalling *n* = 2542 over 20 days [26, 28]. Some focus on the number of clinics involved. While most publications mention the number of clinics, many fail to specify the number of operatories within each clinic. Only five publications report the number of patients treated during the sampling period [5, 26, 27, 29, 33]. The duration of these studies also differs, ranging from a single-day audit [33], to multi-month observations [29]. The primary data collected from all studies are the masses of polymer waste, although additional details, such as waste per compound, were scarce [30]. Notably, the study by Martin et al. documented 150 observations by auditors over 120 days across three different types of clinics: a teaching hospital, an NHS primary care clinic, and two private primary clinics [5] (Table 2).

**Table 2:** Characteristics of waste audits that include polymer waste data.

Study	Setting	Sample size	Time	Data
Yeoh et al. [33]	1 dental faculty undergraduate clinic, 50 patient units/students	50 patients	1 day, 2021.08	Polymer waste mass
Oxborrow et al. [32]	1 dental faculty undergraduate preclinic, simulated restorative intervention	No patients, 72 students	4 h, 2023.02.22	Polymer waste mass
Martin et al. [5]	4 clinics, 1 teaching hospital + 1 NHS primary care+ 2 primary/private care	150 observations (Patient *n*, no information)	120 days, 2019.10-2020.02	Polymer waste mass
Alstrom [31]	1 clinic, private, 4 dentist+3 hygienist operatories	Patient *n*, no information	5 days, 2021.04-2021.04	Polymer waste mass
Wyssusek et al. [30]	1 hospital, 22 operating rooms	5 patients/day (estimated)	31 days, 2019.08-2019.09	Polymer waste mass per compound
Aghalari et al. [29]	53 clinics, 40 general dentistry + 13 speciality, randomly selected	Patient *n*, no information—(Average 240 pts./day (gen) 6 78 pts/day (spec))	3 days/week for one month, 2018.06-2018.08	Polymer waste mass
Voudrias et al. [28]	20 clinics, 19 private + 1 public, randomly selected,	2542 patients	20 days, 2013.04-2013.07	Polymer waste mass
Momeni et al. [27]	48 clinics, partaking from *n* = 60 invited	Patient *n*, no information	3 days/week for two months, 08.2017-09.2017	Polymer waste mass
Mandalidis et al. [26]	20 clinics, 19 private + 1 public, randomly selected	2542 patients	20 days, 2013.04-2013.07	Polymer waste mass
Majlesi et al. [25]	21 clinics, public, randomly selected,	Patient *n*, no information	3 days, 2016.06-2016.09	Polymer waste mass
Richardson et al. [24]	1 clinic, mixed NHS/private dental practice	Patient *n*, no information	2 days x 2, 2014.08 2014.09	Polymer waste mass
Amouei et al. [23]	25 clinics, 20 general dentistry + 5 speciality clinics	Patient *n*, no information	3 samples, year no information	Polymer waste mass
Nabizadeh et al. [22]	50 clinics, 45 public + 5 private, randomly selected	Patient *n*, no information	3 days, 2012	Polymer waste mass
Nabizadeh et al. [21]	28 clinics, 10 general dentistry + 8 speciality + 5 ‘practical dentists’ + 5 denturist, randomly selected	Patient *n*, no information	3 samples, year no information	Polymer waste mass
Kizlary et al. [20]	23 clinics, 20 private + 1 public	Patient *n*, no information	22 days, 2002.05-2002.06	Polymer waste mass
Ozbek et al. [19]	1 dental faculty clinics, 8 departments	Patient *n*, no information—(Average >300 patients/day)	1 day, 2002.04 + 1 working day, 2002.05	Polymer waste mass

#### Risk of bias

Waste audits are conducted for various reasons, and ethical approval is not always required. Some identified reports cited national regulations, while others underscored the necessity for logistical planning within local communities. Several waste audit studies explored best practices, which may require ethical approvals in certain countries. Most reports did not disclose their funding sources; however, some indicated that they received funding from universities and the public. The scores from the eight questions, which assess the potential risk of bias, ranged from 2/8, signifying a high risk, to 5/8. Concerns regarding potential bias were because of i) a failure to segregate monomaterial waste from layered material waste, ii) a lack of segregation between non-polymerised material and polymerised polymeric materials, and iii) the inability to distinguish the quantities of different hard and soft polymers, including HDPE, LDPE, PET, PP, PS, PVC, SR, and elastomers. Two studies were evaluated against fewer criteria because of their particular study objectives [5, 32] (Table 3).

**Table 3:** The potential risk of bias of waste audits that include polymer waste data.

Study	Ethics	Funding	ROB Score (see below^a^)
Yeoh et al. [33]	Approved by the James Cook University Human Ethics Committee (#H8154)	None declared	3/8
Oxborrow et al. [32]	None declared	None declared	3/6
Martin et al. [5]	Approved by the University of Sheffield, School of Clinical Dentistry Ethics Committee.	Public funding, Plastic Research Innovation Fund (EP/S025200/1) & Grantham Centre for Sustainable Futures (U. Sheffield)	3/3
Alstrom [31]	None declared	None declared	4/8
Wyssusek et al. [30]	None declared	Self-funded	2/8
Aghalari et al. [29]	None declared	University funding	4/8
Voudrias et al. [28]	None declared	None declared	4/8
Momeni et al. [27]	Approved by the Ethics Committee, Birjand University of Medical Sciences (Ir.bums.REC.1396.72).	University funding	5/8
Mandalidis et al. [26]	None declared	None declared	3/8
Majlesi et al. [25]	None declared	University funding	5/8
Richardson et al. [24]	Approved by Plymouth University’s Faculty of Health and Human Sciences Research Ethics Committee.	University funding	2/8
Amouei et al. [23]	None declared	None declared	5/8
Nabizadeh et al. [22]	None declared	None declared	4/8
Nabizadeh et al. [21]	None declared	University funding	3/8
Kizlary et al. [20]	None declared	None declared	2/8
Ozbek et al. [19]	None declared	None declared	2/8

	2024 Yeoh	2024 Oxborrow	2022 Martin	2021 Alstrom	2020 Wyssusek	2020 Aghalari	2018 Voudrias	2018 Momeini	2018 Mandalid	2018 Majlesi	2016 Richards	2016 Amouei	2014 Nabizad	2012 Nabizad	2005 Kizlary	2004 Osbek
1. Are sampling processes explained?	Y	Y	Y	Y	N	Y	Y	Y	N	Y	Y	Y	Y	Y	N	N
2. Are methods used for measuring mass explained?	Y	Y	Y	Y	Y	Y	Y	Y	Y	Y	Y	Y	Y	Y	N	N
3. Are calibration procedures explained?	N	Y	Y	N	N	N	N	Y	N	Y	N	Y	Y	N	N	N
4. Were receptacles utilised to segregate waste?	N	n.a	n.a	Y	N	Y	Y	Y	Y	Y	N	Y	Y	N	Y	Y
5. Was biomedical waste separated from other types of waste?	Y	n.a	n.a	Y	N	Y	Y	Y	Y	Y	N	Y	N	Y	Y	Y
6 Was polymeric single-material waste differentiated from composite material waste?	N	N	n.a	N	N	N	N	N	N	N	N	N	N	N	N	N
7. Was non-polymerised material separated from polymerised polymeric materials?	N	N	n.a	N	N	N	N	N	N	N	N	N	N	N	N	N
8. Were different polymers (PETE, HDPE, PP, PVC, LDPE, PS, SR, elastomers) distinguished?	N	N	n.a.	N	Y	N	N	N	N	N	N	N	N	N	N	N
Sum score (maximum score: 8)	3	3/6	3/3	4	2	4	4	5	3	5	2	5	4	3	2	2

*n.a.* not apply.

^a^ROB score details.

#### Results of individual studies

Numerous studies failed to provide estimates of the mean mass of polymer waste per patient, procedure, day, and year. These estimates had to be derived from other data presented within the papers. The polymer waste per patient information is sourced from six studies [5, 26, 28, 29, 32, 33]. Estimates of the waste attributed to specific items per patient are ~81 g of aprons [33], 4 to 56 g of gloves [26, 28, 29, 32, 33], 13 to 24 g of impression materials [32, 33], 3 to 17 g of masks [32, 33], and 3 g of suction tips [29]. A substantially higher estimate of polymer waste per patient is 384 g, due to discarded single-use items [5]. Nine studies provided data to enable estimates of polymer waste per operatory room per day [5, 19, 20, 24, 26, 28–30, 33]. The amount of waste in hospital operating theatres is not directly transferable to oral healthcare clinics [30], but PET predominates, which has also been identified as the primary aerosol contaminant in oral healthcare clinics [41].

The estimated total annual mass of SUP waste from 47,000 oral healthcare professionals in the United Kingdom who perform operative procedures on five patients per day for 160 days per year is 14.4 tonnes [5]. If additional COVID-19 protection is required for the clinic staff, the estimate becomes 27 tonnes [5]. With these premises, a single professional generates 307.2 kg of SUP waste per year, 1.92 kg per day, and 384 g per patient. If additional COVID-19 protection for the working staff is required, the estimates are 575.2 kg per year, 3.6 kg per day and 719 g per patient. The estimates surpass those based on waste audits conducted in Iran [21–23, 25, 27] and the USA [30].

Considering the various study designs, potential biases, and evaluated outcomes, we found it unfeasible to combine the data in a meta-analysis (Table 4).

**Table 4:** Results of waste audits that include polymer waste data.

Study	Source^a^	Polymer waste per patient (g)	Polymer waste per operatory per day (kg)	Polymer waste per operatory per year (kg)
Yeoh et al. [33]	Table 1	81 apron 56 gloves 49 plastics other 17 mask	not reported	not reported
Oxborrow et al. [32]	Table 1 div. *n* = 72 students	213 plastic 24 impression material 22 gloves 13 impression tips 3 masks	not reported	not reported
Martin et al. [5]	Per year: Table 5 divided by 47k dentists, per day: divided by 160 workdays, per pts: divided by 5 patients per day	384 plastic single-use items 719 plastic single-use items COVID-19 extra	1.9/3.8 (5/10 patients/day) 3.6/7.2 (5/10 patients/day) COVID-19 extra	307 (160 workdays x 5 patients/day 575 (160 workdays x 5 patients/day COVID-19 extra
Alstrom [31]	Fig. 1. Convert from lbs & 7 units	not reported	not reported	(48 working weeks) 16 gloves 12 Plastic cups, suction air-water tips 10 Barriers & sterilisation bags 2 masks
Wyssusek et al. [30]	141 kg PET = 40% of total, div. per day & per 22 operatories	not reported	0.29 Polyethylene terephthalate (PET) 0.17 Polyvinylchloride (PVC) 0.15 Polypropylene (PP) 0.07 Copolymers (PP/PE) 0.04 Polyurethane (PU)	not reported
Aghalari et al. [29]	Tables 3 and 4, div. aver. 40/13 GPs & Spec.	7,3 nylon (non-infectious) 6,5 other plastics (non-infectious) 4.0 glove (infectious) 3,3 suction tips (infectious)	0.03 nylon (non-infectious) 0.03 other plastics (non-infectious) 0,02 glove (infectious) 0.01 suction tips (infectious)	not reported
Voudrias et al. [28]	Table 2 aver. dentist education	14 gloves 7 other plastic items	0.10 gloves 0.05 other plastic items	not reported
Momeni et al. [27]	Table 1 sum div. *n* = 48 clinics	not reported	not reported	26 gloves 6 other plastic items 5 syringe tips
Mandalidis et al. [26]	Table2	15,2 Gloves 7 other plastic items	0,12 gloves 0,05 other plastic items	not reported
Majlesi et al. [25]	Table 2 div. *n* = 22 clinics	not reported	not reported	2 gloves 1 suction tips
Richardson et al. [24]	Fig 4. div. 5 days	not reported	0,35 nitrile gloves 0,11 plastic 0,11 separable paper plastic	not reported
Amouei et al. [23]	Table 3, div. aver. 20/5 GPs & Spec.	not reported	not reported	99 Gloves (Infectious) 45 Nylons (non-infectious) 31 Plastic (non-infectious) 29 Suction head (Infectious)
Nabizadeh et al. [22]	Table 2, div. aver. 45/5 public/private clinics	not reported	not reported	19 Gloves (Infectious) 11 Nylons (non-infectious) 4 Plastic (non-infectious) 4 Suction head (Infectious) 2 Syringes plastic (Infectious)
Nabizadeh et al. [21]	Table 2, div. aver. 143/25 public/private clinics	not reported	not reported	163 gloves 119 suction 109 nylon 356 ‘nylons’ (material types)
Kizlary et al. [20]	Table 2	not reported	0,13 latex/PVC gloves 0,07 plastic-coated paper (infectious) 0,05 plastic (infectious) 0,03 saliva ejectors 0,02 elastomer impression	not reported
Ozbek et al. [19]	Table 2 & 3, aver.	not reported	0,13 rubber gloves	not reported

Average mass of polymer waste per patient (g), day (kg) and year (kg).

^a^Original publication figure or table showing the extracted data

### Polymer pollution into air and waterways

On-site sampling studies of polymer pollution in oral healthcare clinics have concentrated on the emissions of polymer NMP into the ambient air during actual clinical interventions [34–36, 38–41]. One study conducted in a hospital clinic contributes to the existing data by describing aerosolised polymer pollution produced in a cardiothoracic surgical operating theatre [37] (Table 5).

**Table 5:** On-site sampling studies of polymer NMP air pollution.

Study	Country	Objectives	Methodology
Akhtar et al. [41]	Pakistan, Lahore	To investigate the indoor source and abundance of microplastics in private dental units during routine professional activities.	Air sampling in an oral healthcare operatory
Tang et al. [40]	China, Shanghai	To quantify ultrafine particle concentrations in real multi-chair dental clinics and to compare the levels of ultrafine particles produced by different dental procedures	Air sampling in an oral healthcare operatory
Rafiee et al. [39]	Canada, Edmonton	To characterise the size and concentrations of particles emitted from seven different dental procedures	Air sampling in an oral healthcare operatory
Lahdentausta et al. [38]	Finland, Helsinki	To measure aerosol generation in various dental procedures in clinical settings.	Air sampling in an oral healthcare operatory
Field et al. [37]	U.K., Kingston	to quantify and characterise the microparticles present within the surgical environment over a one-week sampling period	Air sampling in an oral healthcare operatory
Polednik [36]	Poland, Lublin	To report comprehensive measurements of aerosol particle number and mass concentrations during individual dental procedures in real working conditions.	Air sampling in an oral healthcare operatory
Sotiriou et al. [35]	USA, Massachusetts	To describe the first systematic measurements of particle number, size, mass concentrations, and elemental composition of the particles in a dental office during dental drilling procedures associated with fillings and crowns	Air sampling in an oral healthcare operatory
Henriks-Eckerman et al. [34]	Finland, Turku	To study exposure to airborne methacrylates and natural rubber latex allergens during the placement of dental resin-based composites in six clinics	Air sampling in an oral healthcare operatory

#### Study characteristics

The study clinics varied markedly and included one open-concept clinic comprising six units [39], one public clinic [38], one hospital operating theatre with an adjoining anaesthetic room [37], and six clinics (three private and three public) featuring between one and seven operatories [34]. The participants and sample sizes ranged from *n* = 10 [39], to *n* = 84 adult patients undergoing 253 procedures [38]. Additionally, the interventions varied, with the dental interventions comprising routine adult dental care [34, 38], or orthodontic bonding and debonding [39]. The analytic technologies included optical particle sizers, mass concentration measurements using laser photometers, personal samplers for microbial analysis, glass sampling beakers analysed via micro-Fourier-transform infra-red spectroscopy, and thermal desorption tubes examined with gas chromatographs. Ultimately, the results comprised particle count (*n*/m³) or particle mass concentrations (ng/m³), the distribution of chemical compounds, and monomer concentration above the patient’s mouth and in the clinician’s breathing zone (Table 6).

**Table 6:** Characteristics of on-site sampling studies of polymer NMP air pollution.

Study	Setting	Participants & sample size	Intervention	Methodology	Outcomes
Akhtar et al. [41]	10 clinics, 5 private + 5 private teaching hospitals	Number of patients not reported	Ordinary patient care, including RBC restorative care	Dust collected from surfaces, treated and extracted for microplastics using sieving, oxidative extraction, density separation, filtration, visual observation and polymer identification by digital microscope and ATR-FTIR spectroscopy	NMP count concentration (n/g dust)) NMP inhalation intake (n/g dust/day and year) NMP morphologies NMP morphologies: chemical compounds
Tang et al. [40]	1 hospital clinic, multiple units	Number of patients not reported	Ordinary patient care, including RBC restorative care	NanoTracer, every 10 s. real-time concentration of NMPs within the range of 10–300 nm. Also, information that may influence the concentration of NMP, including the number of patients and the dental procedures	NMP count concentration (n/cm³)
Rafiee et al. [39]	1 clinic, 6 units, open-area concept	10 patients receiving either orthodontic bonding or debonding and denture adjustment)	Different treatment procedures, of which 3 included RBC. 20 min preprocedural ->40 min during procedure ->20 min postprocedural	Optical NMP sizer + mass concentration measured with a DustTrack laser photometer + personal sampler fixed to the clinician faceshield for microbial analysis	NMP count concentration (not reported) NMP mass concentration (not reported) NMP size distribution, 13 sizes (0.3–10 μm)
Lahdentausta et al. [38]	1 clinic, public	84 regular adult patients undergoing 253 procedures	Ordinary patient care, including RBC restorative care	Optical NMP sizer measuring NMPs 0.3 to 10 μm, 50 cm away from patient mouth. NMP mass concentrations were calculated by converting volume size distributions to mass size distributions, assuming an NMP density of 1 g/cm³.	NMP count concentration (n/cm³) NMP mass concentration (μg/m³) NMP size distribution (<1, 1–5, >0.5 μm)
Field et al. [37]	1 hospital, operating theatre + adjoining anaesthetic room	Number of patients not reported	Standard cardiothoracic surgery, including coronary artery bypass grafts, lobectomies, and surgical tracheostomies.	Glass sampling beakers size 1 L with a known base surface area. 8 a.m.–8 p.m.–8 a.m. repeated x7. Beakers were rinsed, filtered onto 0.02 μm membranes and analysed using micro-Fourier-transform infra-red spectroscopy.	NMP count n/m²/day Mean (SD) – maximum NMP size (μm) Prevailing NMP morphology: Prevailing compounds:
Polednik [36]	1 clinic	Number of patients not reported	Ordinary patient care, including RBC restorative care	Ultrafine NMP counters, optical spectrometers, NanoTracer, aerosol monitors, inductively coupled plasma optical emission spectrometry, carbon analyser, and passive sampling using Petri plates.	NMP count concentration (n/cm³) NMP mass concentration (μg/m³) NMP size distribution (<0.5 and >0.5 μm)
Sotiriou et al. [35]	1 clinic	Number of patients not reported	Ordinary patient care, including RBC restorative care	Continuous NMP measurements taken every minute using Climet, PTRAK, and DustTrak Integrated NMP samples were collected for about 5 h on each of five sampling days using PM2.5 and PM10 samplers	NMP count concentration (n/cm³) NMP mass concentration (μg/m³) NMP size distribution (<0.5 and >0.5 μm)
Henriks-Eckerman et al. [34]	6 clinics, 3 private + 3 public, 1-7 units	Number of patients not reported	Ordinary patient care, including RBC restorative care	Thermal desorption tubes containing Tenax TA, above the patient’s mouth, at the nurse’s breathing zone, gas chromatograph (DL < 0.5 ng/m³)	Monomer concentration above the patients’ mouth, at the nurse’s breathing zone and in the sampling area (mg/m³)

*ATR-FTIR* attenuated total reflectance fourier transform infra-red spectroscopy, *DL* detection limit, *NMP* nano-microsize particle, *RBC* resin-based composite.

#### Risk of bias

The potential risk of bias was considered low for two studies [34, 37]. The other two papers lacked detailed information regarding the calibration process for the analytical equipment and were therefore considered to have a low to moderate risk of bias [38, 39] (Table 7).

**Table 7:** Risk of bias of on-site sampling studies of polymer NMP air pollution.

Study	Ethics	Funding	ROB Score (see below*)
Akhtar et al. [41]	Not applicable	Self-funded	2/3
Tang et al. [40]	None declared	Public: Shanghai 3-year Public Health Action Plan (GWV-10.1-XK11)	2/3
Rafiee et al. [39]	Approved by the University of Alberta ethics committee (Pro00103510)	Public: American Academy of Orthodontics Fund (AAOF BRA)	2/3
Lahdentausta et al. [38]	Approved by the local ethical committees of Helsinki University Hospital and the Helsinki City (HUS/1701/2020, HUS/1450/2020, HEL 2020–007596T130201)	Public: Suomen Naishammaslaakarit + Helsinki and Uusimaa Hospital District + Finska Lakaresallskapet + Rauha Ahokas Fund + Academy of Finland	2/3
Field et al. [37]	Approved by the Faculty of Science and Engineering Ethics Committee, reference number FEC_2020_106.	Public: Health Education England	3/3
Polednik [36]	None declared	Public: Polish National Science Centre under Grant NCN 7498/B/T02/2011/40)	3/3
Sotiriou et al. [35]	None declared	University funding	3/3
Henriks-Eckerman et al. [34]	None declared	None declared	3/3

	2022 Akhar	2022 Tang	2022 Rafiee	2022 Lahdentausta	2022 Field	2014 Polednik	2008 Sotiriou	2001 Henriks-Eckerman
Are the details of the sampling method described adequately?	Y	Y	Y	Y	Y	Y	Y	Y
Are the details of the analytical methodology described adequately?	Y	Y	Y	Y	Y	Y	Y	Y
Are the details of the calibration process for the analytic equipment adequately described?	N	N	N	N	Y	Y	Y	Y
	2	2	2	2	3	3	3	3

^* ROB criteria.^

#### Results of individual studies

Working with polymer materials in patients’ mouths releases NMPs containing 2-HEMA and TEGDMA, with the highest concentrations found just above the patients’ mouths. Still, these rapidly dilute further from the working area [34]. Hospital surgeons are exposed to atmospheric NMPs, with the predominant compounds being volatilised PET, PP, PE, and nylon [37]. The quantity and dimensions of NMPs in dental clinics are influenced by the type of rotating burs and other work instruments clinicians use [38]. Ultimately, the quantity of NMPs increases during work procedures but may surge even higher after the procedures are completed [39] (Table 8).

**Table 8:** Results of on-site sampling studies of polymer NMP air pollution.

Study		Quantity measures			
Akhtar et al. [41]	NMP count (n/m²) NMP: Mean (SD) Inhalable NMP (<10 μm): Mean NMP morphologies NMP chemical compounds NMP morphologies: chemical compounds	Private clinics	Teaching hospital		
		587 (185)	1083 (134)		
		20/day - 5260/year	29/day - 765/year		
		Beads 0–1%; Films/sheets 6–22%; Fragments 16–36%; Foam 0–1%; Fibres 48–74% PET: 39%, PP: 28%, LDPE 11%, Nylon 8%, PTFE 7%, EVA 5%, Other 2% Beads: PS 85% PUR 11%; Films/sheets: LDPE 24%, PET 19%, PTFE 11%; Fragments: LDPE 43%, PP 25%, PS 21%; Fibres: PET 50%, LDPE 28%, PP 18%			
Tang et al. [40]	NMP count concentration (n/cm³) Clinis working hours, mean (SD) Clinic work associated with RBC activities, mean (SD)	10,057 (5725)	13,695 (5518) – ratio 1.43	- maximum 62233 - 1.87	
Rafiee et al. [39]	NMP count (n/mL)/mass (mg/m³)	Pre-grinding	Grinding	Post-grinding	
	Denture adjustment: count/mass	5400/0.01	- 8600/0.01	- 9200/0.01	
	Orthodontic bonding: count/mass	600/0.01	- 700/0.01	- 8600/0.01	
	Orthodontic debonding count/mass	200/0.01	- 5900/0.02	- 2700/0.01	
Lahdentausta et al. [38]	NMP count (n/cm³)/mass (μg/m³))	Air turbine	Low-speed handpiece	High-speed handpiece	
	<PM_1_ (<1 μg/m³) count/mass	3.3/0.06	2.6/0.06	1.4/0.03	
	PM_1_–PM_5_ (1–5 μg/m³) count/mass	0.1/0.4	0.06/0.2	0.05/0.1	
	>PM_5_ (>5 μg/m³) count/mass	0.003/0.2	0	0	
	Total PM: count/mass	3.4/1.0	2.7/0.3	1.5/0.15	
Field et al. [37]	NMP count (n/m²/day)	Operating Theatre	Anaesthesia room:		
	Mean (SD) – maximum	1924 (3105) − 9258	541 (969) − 3368		
	NMP mean size (μm)	30 × 92			
	Prevailing NMP morphology:	Fragmented-shape (78%);			
	Prevailing chemical compound:	PET (37%) PP (25%) PE (7%) Nylon (13%)			
Polednik [36]	NMP count & mass, ratios versus background	Grinding composite	All other		
	Count PN_0.01–0.3_ Mean (SD), median, maximum	6.2 (5.6) 4.2/31.8	2.8 (1.8) 2.3/31.8		
	Count PN_0.02–1_ Mean (SD), median, maximum	16.1 (11.4) 12.4/66.7	4.8 (4.2) 3.4/66.7		
	Count PN_0.3–2_ Mean (SD), median, maximum	1.6 (0.2) 1.5/2.2	1.1 (0.8) 1.0/5.9		
	Count PN_>2_ Mean (SD), median, maximum	7.0 (4.6) 5.4/22.1	2.9 (2.8) 1.7/32.5		
	Mass PM_1_ Mean (SD), median, maximum	3.4 (1.4) 3.1/7.7	1.9 (1.3) 1.6/8.4		
	Mass PM_2.5_ Mean (SD), median, maximum	3.7 (1.5) 3.4/6.4	2.0 (1.4) 1.7/9.2		
	Mass RESP Mean (SD), median, maximum	4.2 (1.9) 3.7/10.3	2.2 (1.6) 1.8/11.1		
	Mass PM_10_ Mean (SD), median, maximum	5.6 (3.1) 5.2/16.0	2.8 (2.2) 2.1/16.2		
	Mass TOTAL Mean (SD), median, maximum	6.1 (3.6) 5.7/18.4	3.0 (2.4) 2.2/18.7		
Sotiriou et al. [35]	NMP count & mass	Background	Clinics over 4 days		
	NMP count concentration (n/cm³)	2129 (294)	8683 (7472)		
	PM_10_ mass concentration (μg/m³) mean	5.2	11.9		
	NMP size distribution (<0.5 and >0.5 μm)	222 and 2.3	232–374 and 2.7–6.5		
Henriks-Eckerman et al. [34]	Monomer compound (ng/m³)		Placement	Finishing	Removal
	Above patient mouth	2-HEMA	13 (68)	−12 (36)	− 4 (5)
	Median (Maximum)	TEGDMA	<1 (16)	−8 (19)	−54 (82)
	Nurse breathing zone	2-HEMA	3 (33)		
	Median (Maximum)	TEGDMA	<1 (15)		
	Area sampling	2-HEMA	<1	(<1)	
	Median (Maximum)	TEGDMA	<1	(<1)	

*2-HEMA* 2-hydroxyethyl methacrylate, *LDPE* low-density polyethylene, *NMP* nano- microsize particle, *PE* polyethylene, *PET* polyethylene terephthalate, *PM*_1__/__2.5__/__5__/__10_ particulate mass smaller than 1/2.5/5/10 μg, *PN*_0.3–0.5/0.5–1/1–2/2–5/5–10/>10_ particulate count within different μg size ranges, *PP* polypropylene, *PS* polystyrene, *PTFE* tetra fluoroethylene, *PUR* Polyurethane, *RBC* resin-based composite, *RESP* respirable particles ≤4 μg, *SD* standard deviation, *TEGDMA* Triethylene glycol dimethacrylate, *UDMA* urethane dimethacrylate.

Any meta-analysis was deemed unsuitable due to the heterogeneity of study designs and outcomes measured.

### Waste landfill disposal and monomeric eluates or polymer NMPs

The potential pollution from waste containing polymers deposited in municipal landfills has been explored only by a limited number of studies by a research group in Sheffield, U.K. [42–47] (Table 9).

**Table 9:** Methodology of studies addressing monomeric eluates and polymer NMPs from waste landfills.

Study	Country	Objectives	Methodology
Mulligan et al. [47]	U.K., Sheffield	To characterise the size and distribution of dental RBC microparticles created from simulated clinical use, (ii) to analyse the effect of their ageing in water to simulate environmental release, and (iii) to assess the potential reactivity of the microparticles in the environment and their potential to become toxic polluters and vectors for pollution.	CAM & non-CAM RBC NMPs aged 12 months in municipal tap water microcosm
Martin et al. [46]	U.K., Sheffield	To assess the environmental pollutant risk of dental RBC after interment of cadavers containing restorations via an in vitro analysis of elution of monomers into groundwater	Ceramic teeth with RBC restorations aged in artificial saliva and stored in groundwater microcosm
Mulligan et al. [45]	U.K., Sheffield	To assess the environmental pollutant risk of the waste from CAD/CAM dental RBC via analytical characterisation of the microparticulate released and monomeric eluates	Fresh & aged CAM RBC NMPs stored in a ‘microcosm simulating environment’
Falyouna et al. [44]	U.K., Sheffield	To extract and quantify the monomers released from dental RBC into landfill leachate and secondarily to characterise the chemical changes of sterilised and non-sterilised landfill leachate in the presence of dental RBCs	RBC specimens stored in sterilised & non-sterilized landfill leachate
Mulligan et al. [43]	U.K., Sheffield	To determine whether incineration of waste dental RBC is a suitable alternative to landfill disposal.	Polymerised & non-polymerised RBC specimens stored in sterilised & non-sterile landfill leachate
Mulligan et al. [42]	U.K., Sheffield	To quantify the potential environmental pollutant effect from the release of microplastics and partly polymerised dental composite components following clinical finishing regimes.	RBC NMPs stored in tap water

*CAM* computer-assisted manufactured, *NMP* nano- and microsize particles, *RBC* resin-based composite.

#### Study characteristics

The characteristics of the various simulation studies conducted by one research group at the University of Sheffield, U.K., were relatively consistent. A common feature is that polymer specimens or NMPs produced from these specimens were stored in solutions mimicking municipal landfill conditions, including incubation in sterilised and non-sterilised landfill leachate [43, 44], ‘microcosms’ storage for three months [45], and groundwater at 10 °C for 12 months [46], or in tap water for 12 months [47]. Various analytical methods, including chromatography, spectrometry, potentiometry, and electron microscopy, were utilised to identify particle compounds, dimensions, and protonation-deprotonation behaviours across multiple pH levels and time points (Table 10).

**Table 10:** Characteristics of studies addressing monomeric eluates and polymer NMPs from waste landfills

Study	Setting	Participants & sample size	Intervention	Analysis	Time	Outcomes
Mulligan et al. [47]	standardised procedure in a simulated environment	Sample *n*, no information	Particulate RBCx2 (Direct place & CADCAM) x2 (commercial & in-house control) added to tap water -> aged 12 months	Scanning electron microscopy, laser diffraction particle size analysis, micro-Fourier transform infra-red spectroscopy and potentiometric titration.	0, 12 months	Particle size + Specific surface area (m²/kg) + pH of zero proton charge (pHzpc)
Martin et al. [46]	standardised procedure in a simulated environment	Sample *n*, no information	Polymerised RBCx2 (commercially available or in-house control) was added to artificial saliva at 35°C -> placed in groundwater at 10°C for 12 months.	Solid phase micro-extraction coupled with high-performance liquid chromatography	0, 1 month, 5, 10, 12 months	Eluted monomers: BPA, TEGDMA, UDMA, HEMA, Bis-GMA, (mg/L)
Mulligan et al. [45]	standardised procedure in a simulated environment	Sample *n*, no information	Particulate RBCx2 (commercially available or in-house control) added to microcosms simulating environmental release scenarios including variable pH -> aged 3 months	Particle size analysis, Fourier-transform infra-red spectroscopy and potentiometry + solid phase micro-extraction coupled with high-performance liquid chromatography	0, 3 days, 3 months	Eluted monomers: BPA, TEGDMA, UDMA, HEMA, Bis-GMA (μg/L) + Protonation-deprotonation behaviour
Falyouna et al. [44]	standardised procedure in a simulated environment	126 samples	Polymerised RBC (0.5 g) was added to sterilised & non-sterilised landfill leachate (14 mL), incubated at 35 °C	Solid phase micro-extraction coupled with high-performance liquid chromatography + pH metre, gas chromatography, ion chromatography and inductively coupled plasma mass spectrometry	0, 1, 3, 7, 14, 28 days	Eluted monomers: BPA, TEGDMA, UDMA, HEMA, Bis-GMA (μg/L) + PH + concentration of methane, carbon dioxide,
Mulligan et al. [43]	standardised procedure in a simulated environment	Sample *n*, no information	Unpolymerized & polymerised RBC x2 (commercially available or in-house control) added to sterilised & non-sterilised landfill leachate, incubated 35 °C -> Incineration ≈850 °C for 2 h,	Gases, ions and compounds quantified via solid phase micro-extraction, high-performance liquid chromatography + gas chromatography, ion chromatography and inductively coupled plasma mass spectrometry	up to 28 days	Eluted monomers: BPA, TEGDMA, UDMA, HEMA, Bis-GMA (μg/L)
Mulligan et al. [42]	standardized procedure in a simulated environment	Sample *n*, no information	Particulate polymerised RBCx2 was added to tap water and sampled at regular intervals	Laser diffraction + high-performance liquid chromatography & solid phase micro-extraction,	up to 1 month	Particle size + Eluted monomers BPA, TEGDMA, UDMA, HEMA, Bis-GMA (ppb)

*Bis-GMA* bisphenol A glycidyl methacrylate, *BPA* bisphenol-A, *COM* commercial RBC product, *HEMA* hydroxyethyl methacrylate, *inH* in-house, *PPB* parts per billion, *RBC* Resin-based composite, *TEGDMA* Triethylene glycol dimethacrylate, *UDMA* Urethane dimethacrylate.

#### Risk of bias

The data from the simulation studies were presented at IADR research meetings [42, 43, 45, 46], and in proceedings from one conference [44]. The possibility of thoroughly assessing all methodological aspects of the experiments is limited; thus, their risk of bias is high when considered individually. However, the results are either summarised or referenced in a single peer-reviewed publication, which has been deemed to have a low risk of bias [47] (Table 11).

**Table 11:** The potential risk of bias of studies addressing monomeric eluates and polymer NMPs from waste landfills

Study	Ethics	Funding	ROB Score (see below*)
Mulligan et al. [47]	Not applicable	Public: Shirley Glasstone Hughes Research Grant (Ref. 002-2013)	3/3
Martin et al. [46]	Not applicable	Public: Shirley Glasstone Hughes Trust Fund	1/3
Mulligan et al. [45]	Not applicable	Public: Shirley Glasstone Hughes Trust Fund	1/3
Falyouna et al. [44]	Not applicable	Public: Shirley Glasstone Hughes Trust Fund & BDA (002-2013)	2/3
Mulligan et al. [43]	Not applicable	Public: Shirley Glasstone Hughes Trust Fund	1/3
Mulligan et al. [42]	Not applicable	Public: Shirley Glasstone Hughes Trust Fund (Ref. 002-2013)	1/3

	2021 Mulligan	2019 Martin (Abstract)	2018 Mulligan (Abstract)	2018 Falyouna (Proceedings)	2017 Mulligan (Abstract)	2015 Mulligan (Abstract)
Are the details of the sampling method described adequately?	Y	Y(½)	Y(½)	Y	Y(½)	Y(½)
Are the details of the analytical methodology described adequately?	Y	Y(½)	Y(½)	Y	Y(½)	Y(½)
Are the details of the calibration process for the analytic equipment adequately described?	Y	N	N	N	N	N
	3	1	1	2	1	1

^* ROB criteria.^

#### Results of individual studies

The experiments carried out by the research group over a decade yield incremental findings. The initial study, conducted in 2015, outlines the leakage of predominantly Urethane Dimethacrylate (UDMA) and Triethylene Glycol Dimethacrylate (TEGDMA) monomeric compounds from resin-based composite (RBC) waste [42]. The subsequent reports describe how aged NMPs exhibit markedly different surface characteristics compared with fresh NMP, as well as compelling data indicating the role of microorganisms in the degradation process and the release of eluates [43–46]. The latest experiments provided further evidence of the likelihood that the behaviour of protonation and deprotonation across the pH range creates surface groups capable of binding at sites involving carboxyl (pK≈3–5), silanol/silica (pK≈6–7), and hydroxyl groups (pK>8) [45, 47] (Table 12).

**Table 12:** Results of studies addressing monomeric eluates and polymer NMPs from waste landfills

Study	Outcomes	Before storage	At 24 h	At 1 week	At ≥2 weeks
Mulligan et al. [47]	RBC particulate: Size (median μm) Specific surface area (m²/kg)				Commercial In-house (12 months ageing) 6.4 9.6 1290 1017
Martin et al. [46]	Eluted monomers: (mg/L)				Monomers: (12 months ageing) TEGDMA 790 HEMA 48 BPA 45 UDMA 12 Bis-GMA 8
Mulligan et al. [45]	Eluted monomers (ppm)			At day 3 (peak values) TEGDMA 13 BPA 5	
Falyouna et al. [44]	Eluted monomers: (mg/L)	Leachate: non-sterile sterile BPA 1000 1000 TEGDMA 300 300 UDMA 100 100 HEMA 800 800 Bis-GMA < 100	Leachate: non-sterile sterile BPA 1300 1500 TEGDMA 1500 1000 UDMA 200 1200 HEMA 350 300 Bis-GMA < 100	Leachate: non-sterile sterile BPA 1500 1400 TEGDMA 300 400 UDMA 100 900 HEMA 250 150 Bis-GMA < 100	(2/4 weeks) (2/4 weeks) ageing Leachate: non-sterile sterile BPA 1900/1400 2100/1200 TEGDMA 200/500 700/500 UDMA 100/800 500/800 HEMA 250/150 300/300 Bis-GMA <100
Mulligan et al. [43]	Eluted monomers				1 month ageing Leachate: non-sterile, sterile (RBC #1:#2) (RBC #1:#2) BPA 2.9x:1 4.6x:1
Mulligan et al. [42]	Particle size (μm) Eluted monomers (ppb)	1–500 (mean 289)			6 months ageing UDMA 0–3000 TEGDMA 100–1800 HEMA 50–450 BPA 0–275 BisGMA 0–150

*Bis-GMA* Bisphenol A glycidyl methacrylate, *BPA* Bisphenol-A, *COM* Commercial RBC product, *HEMA* Hydroxyethyl methacrylate, *inH* In-house RBC, *RBC* Resin-based composite, *TEGDMA* Triethylene glycol dimethacrylate, *UDMA* Urethane dimethacrylate.

## Discussion

### Limitations of the evidence

#### Potentially biased data estimates

We identified a significant potential bias using our custom scale, which should not be construed as a criticism of the reported waste audit procedures. In most countries, national regulatory policies govern waste audits across various sectors, emphasising societal needs and requirements. Waste is typically categorised into five fractions: infectious waste, domestic-type waste, toxic chemicals, sharps, and other hazardous waste. Identifying the proportion of polymer waste relative to the total waste amount is challenging and necessitates secondary calculations of the presented data in the original primary studies (Table 4). To better estimate the potential environmental impacts of polymer waste, data should differentiate between aspects such as monomaterial polymers (recyclable) versus layered materials (seldom recyclable), polymerised versus unused non-polymerised scrap materials, and ideally, provide separate information on different polymers, including HDPE, LDPE, PET, PP, PS, PVC, synthetic rubbers and elastomers.

The most common method for quantifying polymer waste involves directly weighing the accumulated waste, provided that end users have adequately segregated the various materials, ideally distinguishing between potentially recyclable items and complex polymers [48]. Random waste selections from storage solutions may be taken from multiple sites or over time to alleviate logistical challenges, provided these are replenished regularly. A more expedient alternative to weighing is to count the number of different polymer items and correlate these counts with known weights. Another option is to complete pre- and post-surgery inventory lists to record waste quantities. One could also argue that reviewing waste consignment or transfer records, or other waste management records or invoice lists, should suffice for estimating polymer waste in comparison to different categories of waste. Which method comes closest to accuracy remains unclear, and ultimately, hospital managers must consider allocating busy healthcare staff to engage in direct patient care rather than other activities [49].

#### Polymer waste reflected by patient demographics and delivered care

Waste generation and management in oral healthcare clinics is a recurring topic in national and international dental and medical journals. However, the number of research papers detailing the qualities and quantities of polymer waste and other waste remains limited. Moreover, the polymer waste produced in oral healthcare clinics will vary significantly depending on the level of care and range of interventions [5]. One must also consider the socioeconomic and cultural contexts of clinician–patient cohorts. Some studies have presented separate analyses of polymer waste generated in public versus private clinics and urban versus rural communities. The heterogeneity of clinical variables renders comparisons between different publications challenging and undermines the validity of any meta-analyses. Unfortunately, there is very little scientific research data available for analysis. A recent exhaustive scoping review concluded that the generalisability of waste audit data within the oral healthcare sector is unreliable, citing several arguments in support of this conclusion [50]. A recent publication from the United Kingdom best represents contemporary clinical practices in developed countries [5]. These estimates were based on a 4-day work week over 40 weeks, with an average of five operative procedures daily.

In Norway, official statistics and research indicate that the average dentist works 230 days a year and treats 10 patients daily [51, 52]. Based on U.K. estimates, the average dentist in Norway generates 883 kg of waste each year solely from polymer SUP refuse. If additional PPE is required during a pandemic, this figure rises to 1654 kg each year.

#### Polymer biomedical or domestic-type waste

A complicating factor in estimating the amount of polymer waste from oral healthcare clinics is that most studies limit their reporting to the mass of hazardous waste compared to domestic-type waste, following guidance from the World Health Organisation [53–55], rather than focusing on the type of material. Furthermore, the packaging of goods and devices has often been overlooked in many of these waste audits, likely leading to an underestimation of the impact of the widespread use of polymer wrapping and barrier foil [5]. The amount of polymer waste can vary by country depending on national legislation that requires mandatory cross-contamination practices. For instance, creating ‘plastic film barriers’ may be compulsory in some countries. Conversely, in others, it may be optional, depending on the circumstances, and even viewed as overly cautious by some [56]. Finally, limited research has documented the outcomes of audits regarding the appropriate waste receptacles set up in clinics and the level of compliance with proper segregation of polymer and other waste, a vital step for ensuring adequate waste stream disposal.

#### Polymer waste management

Managing polymer waste at local, national, and international levels has a significant impact on the global environmental footprint of the oral healthcare sector. Depending on local demographics, the logistics infrastructure for effective polymer waste collection and management may be centralised or decentralised [57]. Regrettably, such deployment remains impractical in numerous countries for several reasons, primarily because optimal technologies are costly and require substantial resources [3]. Open waste burning continues to be practised in various parts of the world, alongside other mismanagement practices that may account for 80–90% of all public waste [58]. The degree to which polymer waste from oral healthcare facilities contributes remains unclear.

#### Elastomer waste proportion of the total waste estimate

Several publications indicate a significant quantity of waste referred to as ‘impression materials’; however, they do not specify the nature of this material [21, 23]. The lack of detail introduces confounding factors since some impression materials are recyclable. For instance, impression plaster and hydrocolloid biopolymers, such as alginate derived from brown seaweed, can be recycled. Conversely, elastomers, made from synthetic polymers, are specifically designed not to degrade. Furthermore, the environmental impacts of the most common dental impression elastomers—condensation silicone, polyether, polysulfide, polyvinyl siloxane, and vinylsiloxanether will likely differ from a comprehensive life cycle perspective. Compounding the difficulty in estimating polymer waste amounts, clinicians often fabricate models from impressions in-house before discarding the impression rather than sending it to a dental laboratory. Estimating elastomer waste is challenging, as impression tray options vary between metal, single-use plastic, and custom-made from a mouldable polymer material. While the former requires manual removal, cleaning, and sterilisation, the latter is typically discarded entirely [59].

#### Product monomeric-polymer contents

Public information on the environmental footprint of manufacturing items containing polymers for the oral healthcare sector is virtually nonexistent. Monomers and polymers that form the basis of products in oral healthcare clinics can be classified as more or less sustainable, according to the ‘green metric’ [48]. Polymers designed for medical use often have significant environmental impacts due to the manufacturing and subsequent refining processes needed to reduce monomer residues and contaminants that could compromise biocompatibility.

### Limitations of the review processes

The data presented in this paper likely reflect publication bias from a planetary perspective, as we included only scientific studies published in English. It is acknowledged that developing countries face the most significant challenges relating to waste management, and data is likely available in publications written in native languages. The authors lacked the multilingual interpretation skills and resources to appraise non-English literature online or in alternative bibliometric databases. We were unable to investigate publication bias and small-study effects due to the limited number of identified studies.

#### Certainty assessment

Despite exhaustive searches, the number of identified records was limited. Although the risk of bias was considered low for many of the studies, the extrapolation to how the measured eluates and NMPs may affect the environment remains uncertain. The heterogeneity of study designs, measurement methodologies, and outcomes prevented the undertaking of meta-analyses. Our pre-hoc choice of at least five studies for undertaking a meta-analysis is because statistical power is low with fewer than five studies [60]. In summary, the current evidence to answer the research question, ‘How much polymer waste and pollution is generated in oral healthcare clinics?’ is limited.

### Implications for practice, policy, and future research

#### Synthetic polymers versus biopolymers

There has been a growing demand for polymers synthesised from sources other than petrochemicals. Biopolymers have emerged as a viable alternative; however, there is limited data on their performance. While some benefits exist in mitigating specific environmental impacts, other challenges remain or are created [61]. Moreover, a notable variation in the biodegradability of products promoted as biopolymers necessitates careful interpretation of the data [62]. Commercial products must demonstrate compliance with standards for the biodegradation of polymers established by the International Organization for Standardization (ISO). The relevant standards for aerobic biodegradation include composting (ISO 14855), soil burial (ISO 17556), and water (ISO 14851 and ISO 14852). The applicable standards for anaerobic biodegradation focus on high solid decomposition (ISO 15985) and water (ISO 14853).

#### Polymers used in cross-contamination practices

Using polymer barrier foils is a legislated practice in certain countries to prevent cross-contamination and optional in others. However, this practice may result in excessive usage and has even been deemed redundant in most circumstances [56].

#### Waste audits in healthcare clinics

The waste audits published in hospital and oral healthcare clinics were challenging to evaluate since the reported data was presented in a generic format or categorised according to descriptors in guidance documents developed by the World Health Organisation [53]. However, these lack the necessary details to enable comparisons of polymer waste across different healthcare clinics. Future studies should adhere to recommendations for best practices and reporting waste audits in healthcare environments [49].

#### Healthcare waste management practices

Numerous papers have outlined best practices for waste management in healthcare clinics [54]. Moreover, innumerable surveys of oral healthcare providers and their staff suggest very high compliance, although opinions vary on the validity of these conclusions. One may also question the use of many non-validated questionnaires and survey designs [63]. Scientists have sought to understand how improved adherence to best waste management practices can be achieved [64]. Focusing on adequate waste segregation and the responsible disposal of each component diminishes environmental impacts and societal costs [65]. Robust advocacy for environmental sustainability within the UK’s oral healthcare sector fosters a sense of optimism about the future [65–67].

One aspect that should be more effectively addressed in healthcare clinics is unwrapping multiple sterile PIDs in preparation for surgery [68]. The practice is required when there is a high chance of adverse events; however, PIDs can be unwrapped sequentially depending on the circumstances during the surgery [69].

#### Waste pollution from PIDs

The data presented by the research group in Sheffield over a decade is compelling evidence that terrestrial microbiomes can degrade polymer waste containing RBC residues and particulates. The first experiment from 2015 emphasised the influence of molecular weight, hydrophilicity, diffusion rates, and the high surface areas of polymer NMPs [42]. The following two reports provide evidence of bacterial-mediated degradation of polymer NMPs in landfill leachate [43, 44]. Even later titles such as ‘Environmental Pollution From the Microparticulate Waste of CAD/CAM Resin-based Composite‘ [45], and ‘Elution of Resin-based Composite Monomers into Groundwaters’ [46], appear to have initiated research elsewhere. The 2021 peer-reviewed publication provided further insights into the degradation of RBC residues and particulates [47]. The research team voiced concerns the following year in the British Dental Journal [70], but neither scientific communities nor the public media appear to have taken extensive action.

Modern wastewater treatment facilities can effectively remove nano- and microparticle-size materials [71]. However, such facilities necessitate significant investments and are energy-intensive, making them unattainable in many parts of the world due to economic constraints [72].

#### Polymer waste management globally

The extent to which global polymer waste is disposed of according to best disposal practices and subjected to pyrolysis, recycling, or reuse remains unknown. Estimates suggest that the proportion of waste from polymer products ending up in private and municipal landfills varies from 25% in Europe, 22% to 43% in the USA, and 66% in India [7, 73]. Unfortunately, additional unknown quantities of hazardous chemicals and compounds are released from uncontrolled open dumpsites to terrestrial ecosystems [74], or inefficient open-burning [75], resulting in the release of persistent organic pollutants into the atmosphere [76], and waterways [72].

#### Polymer waste and pollution likely impact human and planetary health

To limit the length of this SR, we have not addressed in further detail the potential hazards posed by polymer waste and degradation products to humans [6, 77], and planetary ecosystems [78]. Additional degradation occurs in marine and terrestrial environments, rendering polymer NMPs potential vectors for health hazards to living organisms [79, 80], and our planet [81, 82]. There is a strong international consensus that there is a need to end plastic pollution, and creating a culture of striving for a circular economy is one means to achieve this objective [83].

## Conclusions

Our understanding of the short- and long-term effects of polymer degradation and monomer elution on human and planetary health is limited. There is no doubt that a consensus exists on the need to mitigate monomeric eluate and polymer NMP pollution; however, multiple challenges remain to be addressed [84]. Oral healthcare professionals must collaborate in transdisciplinary research with environmental researchers, policymakers, and other stakeholders to identify knowledge gaps and develop sustainable solutions to minimise the environmental impact of polymer waste within the oral healthcare sector.

## Supplementary information

PRISMA Checklist

Supplementary Table 1.

Supplementary Table 2.

Supplementary Table 3.

## Data Availability

Data are provided within the manuscript or supplementary information files.
