# Supplementary material for: Polymer waste and pollution in oral healthcare clinics: a systematic review
Source: BDJ Open. 2025 May 25;11:52. doi: 10.1038/s41405-025-00342-8 (PMC12103492; doi:10.1038/s41405-025-00342-8)
Supplement: Supplementary file 2 — Supplementary Table 1. [file 41405_2025_342_MOESM2_ESM.docx]

Supplementary table 1. Monomeric compounds in polymer items and devices in oral healthcare clinics

| **Compound** | **acronym** | **Dominant compound in items and devices** |
| --- | --- | --- |
| Acrylonitrile Butadiene Styrene | ABS | Container, handle, suture, tray, orthodontic |
| Bisphenol A glycidyl methacrylate / AKA Bowen monomer | Bis-GMA | Dental restoration |
| Chloroprene Rubber / AKA Neoprene | CR | Dressing, elastic, glove, washer |
| Condensation silicone | CS | Impression material |
| Cyanoacrylate | CA | Wound adhesive |
| Ethylene-vinyl acetate / AKA poly(ethylene-vinyl acetate | EVA/PEVA | Soft splint |
| High-Density Polyethylene, | HDPE | Brush, liner, mixing tip, tube, syringe, visor |
| Hydroxyethyl methacrylate | HEMA | Dental restoration |
| Low-Density Polyethylene | LDPE | Bag, barrier film, cover, drape, dressing, package |
| Mixed plastics | MP | Lid, package, tube, 3Dprint |
| Nitrile Butadiene Rubber | NBR | Dressing, elastic, glove, washer |
| Polyamide | Nylon | Bag, drape, dressing, toothbrush |
| Polycarbonate | PC | Model, tray, tube, orthodontic |
| Polycarbonate-modified bis-GMA | PC-bisGMA | Orthodontic, prosthesis, vacuum splint/ retainer |
| Polyester (non-woven) | PES | Disinfectant wipe |
| Polyether | PE / PEt | Impression material |
| Polyethylene terephthalate | PET | Drape, glove, packaging, orthodontic |
| Polyethylene | PE / PEE | Glove, packaging, pouch, strip, syringe, tube |
| Polyisoprene | PI | Glove |
| Polylactic acid | PLA | 3DPrint, customised tray |
| Poly(methyl methacrylate) | PMMA | Orthodontic, prosthesis, mouthguards/splints/ retainer, customised trays |
| Polypropylene film / AKA "Prolene" | PP | Bag, drape, gown, hood, mask, packaging, wraps |
| Polystyrene | PS / PSy | Packaging, prosthesis (alternative re. allergy) |
| Polysulfide / AKA Mercaptan | PSR / PSS | Impression material |
| Polytetrafluoroethylene | PTFE | Membrane (“GBR”), implant screw hole, spacer |
| Polyvinyl chloride | PVC | Bowl, drape, tube, orthodontic, prosthesis, vacuum-formed mouthguard/splint/ retainer |
| Polyvinylidene fluoride | PVDF | Orthodontics |
| Polyvinyl siloxane / AKA Vinyl polysiloxane | PVS / VPS | Impression material |
| Triethylene glycol dimethacrylate | TEGDMA | Dental restoration |
| Urethane dimethacrylate | UDMA | Dental restoration |
| Vinylsiloxanether | VSXE | Impression material |
