# Supplementary material for: Polymer waste and pollution in oral healthcare clinics: a systematic review
Source: BDJ Open. 2025 May 25;11:52. doi: 10.1038/s41405-025-00342-8 (PMC12103492; doi:10.1038/s41405-025-00342-8)
Supplement: Supplementary file 3 — Supplementary Table 2. [file 41405_2025_342_MOESM3_ESM.docx]

Supplementary table 2. Search strategies

| Pubmed  <https://pubmed.ncbi.nlm.nih.gov/> | (Dentist OR dental health services[mesh] OR Dentistry[mesh]) AND (Polymers[mesh] OR Organic Chemicals[mesh] OR plastic*[tw] OR polymer*[tw] OR Resin*[tw] OR acryl*[tw]) AND ((Medical Waste[MeSH] OR Medical Waste Disposal[MeSH] OR Waste Management[MeSH] OR Waste Disposal Facilities[MeSH] OR Hazardous Waste[MeSH] OR Environmental Pollution[MESH] OR Dental Waste[MESH])) |
| --- | --- |
| Cochrane Library  <https://www.cochranelibrary.com/search> | (Dentist OR “dental health services” OR Dentistry) AND (Polymers OR Organic Chemicals OR plastics OR polymer* OR Resin* OR acrylic) AND (“Medical Waste” OR “Medical Waste Disposal” OR “Waste Management” OR “Waste Disposal Facilities” OR “Hazardous Waste” OR “Environmental Pollution” OR “Dental Waste”) |
| Embase (Ovid)  <https://www.embase.com/landing> | (Dentist OR exp dental health services/ OR exp Dentistry/) AND (exp Polymers/ OR exp Organic Chemicals/ OR plastic*.mp. OR polymer*.mp. OR Resin*.mp. OR acryl*.mp.) AND ((exp Medical Waste/ OR exp Medical Waste Disposal/ OR exp Waste Management/ OR exp Waste Disposal Facilities/ OR exp Hazardous Waste/ OR exp Environmental Pollution/ OR exp Dental Waste/)) |
| (EBSCOhost) (ERIC, CINAHL, Risk Management Reference Center, GreenFILE, MEDLINE, eBook Open Access (OA) Collection, AMED - The Allied and Complementary Medicine Database  <https://www.ebsco.com/products/ebscohost-research-platform> | (Dentist OR (MH dental health services+") OR (MH Dentistry+")) AND ((MH Polymers+") OR (MH Organic Chemicals+") OR plastic* OR polymer* OR Resin* OR acryl*) AND (((MH Medical Waste+") OR (MH Medical Waste Disposal+") OR (MH Waste Management+") OR (MH Waste Disposal Facilities+") OR (MH Hazardous Waste+") OR (MH Environmental Pollution+") OR (MH Dental Waste+"))) |
| ScienceDirect  <https://www.sciencedirect.com/> | (Dentist OR "dental health services" OR Dentistry) AND (Polymer OR plastic OR Resin OR Acrylic) AND (environmental pollution OR dental Waste) |
| Web Of Science  <https://www.webofscience.com/> | (Dentist OR dental health services" OR Dentistry) AND (Polymers OR Organic Chemicals" OR plastic* OR polymer* OR Resin* OR acryl*) AND (("Medical Waste" OR Medical Waste Disposal" OR Waste Management" OR Waste Disposal Facilities" OR Hazardous Waste" OR Environmental Pollution" OR Dental Waste")) |
| ProQuest Dissertations & Theses Global  <https://www.proquest.com/education/> | (Dentist OR dental health services" OR Dentistry) AND (Polymers OR Organic Chemicals" OR plastic* OR polymer* OR Resin* OR acryl*) AND ("Medical Waste" OR Medical Waste Disposal" OR Waste Management" OR Waste Disposal Facilities" OR Hazardous Waste" OR Environmental Pollution" OR Dental Waste") |
