# Supplementary material for: Polymer waste and pollution in oral healthcare clinics: a systematic review
Source: BDJ Open. 2025 May 25;11:52. doi: 10.1038/s41405-025-00342-8 (PMC12103492; doi:10.1038/s41405-025-00342-8)
Supplement: Supplementary file 4 — Supplementary Table 3. [file 41405_2025_342_MOESM4_ESM.docx]

**Supplementary table 3. Risk of Bias of waste audits detailing estimates of polymer waste**

YES / NO / UNCLEAR

1. Are waste sampling processes explained? ☐ ☐ ☐

2. Are methods used for measuring waste mass explained? ☐ ☐ ☐

3. Are calibration procedures explained? ☐ ☐ ☐

*4. Is polymer waste segregated in distinctly identifiable receptacles? ☐ ☐ ☐

*5. Is polymer biomedical waste segregated from other types of waste? ☐ ☐ ☐

*6. Is polymer waste of single materials segregated from composite materials? ☐ ☐ ☐

*7. Is non-polymerised segregated from polymerised materials? ☐ ☐ ☐

*8. Are different polymers (PETE, HDPE, PP, PVC, LDPE, PS, SR, elastomer) segregated? ☐ ☐ ☐

* Adapted from: GOV.UK. Healthcare waste: appropriate measures for permitted facilities.
